# A Colour Atlas of the Surgery and Management of Intestinal Stomas

**Published:** 1987-08

**Authors:** M. G. Wilson


M. G. Wilson

### Book Review

**A COLOUR ATLAS OF THE SURGERY AND MANAGEMENT OF INTESTINAL STOMAS**

**L. R. Celestin**

**Wolfe Medical Publications Ltd. Price £32**

This beautifully produced Atlas is a sort of 'guided tour' of intestinal stomas. It deals pictorially with the whole subject with the aid of excellent colour photographs, charts and diagrams, supplemented where necessary with explanatory text. Every aspect of the subject is touched upon starting with the general principles, indications and pathology. Major emphasis is on the technical aspects of constructing stomas but detailed consideration is given to the choice and use of appliances, the complications of the surgery and the problems caused by the stomas themselves.

This book is intended to assist not only the surgeons who make the stomas but also nurses and stoma therapists who service them. The young surgeon forming his own repertoire of techniques will find a wealth of ideas and a sound basis from which to begin. The stoma therapist also will find much useful information in the sections on appliances, skin protectives and deodorants, the action of drugs and dietary considerations.

This excellent, if expensive book conveys its useful message directly and quickly. It is a 'bench book' which needs to be kept on the ward or the operating theatre and should be purchased by hospitals for reference by their staff when the occasions arise.

M. G. Wilson

